# Biocontrol of Mycotoxigenic Fungi by Actinobacteria

**DOI:** 10.3390/jof11010004

**Published:** 2024-12-24

**Authors:** Louise Maud, Nathalie Barakat, Julie Bornot, Selma P. Snini, Florence Mathieu

**Affiliations:** Laboratoire de Génie Chimique, Université de Toulouse, CNRS, INPT, UPS, 31326 Toulouse, France; louise.maud@toulouse-inp.fr (L.M.); barakatnathalie@gmail.com (N.B.); julie.bornot@toulouse-inp.fr (J.B.)

**Keywords:** actinobacteria, biocontrol, mycotoxin, fungi

## Abstract

Actinobacteria are well known for their production of metabolites of interest. They have been previously studied to identify new antibiotics in medical research and for their ability to stimulate plant growth in agronomic research. Actinobacteria represents a real source of potential biocontrol agents (BCAs) today. With the aim of reducing the use of phytosanitary products by 50% with the different Ecophyto plans, a possible application is the fight against mycotoxin-producing fungi in food matrices and crops using BCAs. To deal with this problem, the use of actinobacteria, notably belonging to the *Streptomyces* genus, or their specialized metabolites seems to be a solution. In this review, we focused on the impact of actinobacteria or their metabolites on the development of mycotoxigenic fungi and mycotoxin production on the one hand, and on the other hand on their ability to detoxify food matrices contaminated by mycotoxins.

## 1. Introduction

### 1.1. Origin, Biology and Repartition of Actinobacteria

Actinobacteria are free-living filamentous bacteria that are broadly spread in both terrestrial and aquatic ecosystems. They mainly colonize soils and roots. However, certain species are also able to develop in water and particularly in the marine environment [1], even if their presence is in reality suspected of being due to soil leaching located near the coast [2]. Previously called actinomycete, this former name derives from the Greek words for ray (*aktis* or *aktin*) and fungi (*mukes*). Traditionally, they were considered as transitional forms between fungi and bacteria. They are Gram-positive, filamentous bacteria, containing a DNA rich in guanine-cytosine bases (G-C) [3]. From a nutritional point of view, actinobacteria are mainly chemoheterotrophic and use various energy sources. Most actinobacteria are mesophilic, although thermophilic species exist [4], and grow mainly at pH levels between 6 and 9. As previously described by Lewin et al. (2016), the origin of the *Actinobacteria* is ancient, dating back to thousands of millions of years. It has been demonstrated through genetic sequencing that *Actinobacteria*, *Cyanobacteria*, and *Deinococcus* share a common ancestor. The lineage *Actinobacteria* eventually diverged into various classes and families, which were able to sporulate and grow as filaments, which was not originally the case [5].

With regards to their classification and their taxonomy, *Actinobacteria* are considered among the largest units of the domain *Bacteria*. The previous *Actinobacteria* classification was based on numerical taxonomy, including phenotypical characteristics. A long time ago, the *Actinobacteria* classification was based on 16S rRNA sequencing [3]. Nevertheless, rRNA sequencing is not enough to differentiate between narrowly similar species. A detailed genetic analysis, such as DNA–DNA hybridization, is required to examine related genera and distinguish them [6,7]. The advances in genetic research and the availability of genome sequencing information made the identification of organism-specific genes at the levels of family and genera. It was therefore possible to conclude that the phylum *Actinobacteria* contains six classes: *Actinobacteria*, *Acidimicrobiia*, *Coriobacteriia*, *Nitriliruptoria*, *Rubrobacteria*, and *Thermoleophilia*. There are 16 orders in the class of *Actinobacteria*. In terms of families, the class of *Actinobacteria* contains 43 out of the 53 families of the phylum, leaving only 10 families to the remaining 5 classes [3,8,9]. Due to their high adaptability to pH and temperature, they colonized many extreme environments. Indeed, acidophilic and halophilic species were found, respectively, in acid soils at pH 3.5 and in salt-rich environments such as marine sediment and mangroves. Xerophiles species were also observed in the Algerian desert, and the presence of psychrophiles species was noticed in the permafrost from the Antarctic, but also in the rhizosphere community of *Colobanthys quitensisi* and *Deschampsia antartica* in this region [10,11].

Among *Actinobacteria*, the *Streptomyces* genus is mainly represented in soils, where it participates in the degradation of organic matter due to its ability to hydrolyze complex molecules such as chitin or cellulose [3]. Particularly studied for its ability to produce molecules of interest, the genome of *Actinobacteria* from the *Streptomyces* genus contains between 50 and 70 biosynthetic gene clusters (BGCs) involved in the production of specialized metabolites. However, the production of these specialized metabolites depends on the growth conditions and the morphology that the filamentous bacteria will take [12,13,14], which sometimes complicates the production of certain antibiotic, antifungal, or even insecticidal compounds under laboratory conditions [15].

### 1.2. Properties of Interest

Actinobacteria are highly significant in biotechnology due to their capacity to produce numerous bioactive metabolites with diverse industrial, medical, and agricultural applications. Research on actinobacteria began in the 1940s to identify new molecules with medical applications, such as antimicrobial and antiparasitic compounds. After many years of research, other application domains of actinobacteria were discovered, such as agronomy, notably due to their capacity to produce antifungal compounds. Regarding this latter property, numerous studies over the past thirty years have aimed to identify potential natural antifungal agents, driven by the emergence of resistance to chemical antifungals [16], with an acceleration of research during the last ten years. Thus, various research studies have focused on strains of actinobacteria, mainly on those of the *Streptomyces* genus, which will be the subject of a dedicated section below.

#### 1.2.1. Medical Applications

##### Antimicrobial Compounds

In the context of the emergence of multi-antibiotic-resistant bacteria, also called “superbugs”, many research studies were carried out to identify new antibiotic molecules from actinobacteria, especially from the *Streptomyces* genus. The first antibiotics discovered were streptothricin, isolated in 1942 from *S. lavandulae*, effective against various Gram-negative and Gram-positive bacteria, and streptomycin, isolated in 1943 from *S. griseus* by Albert Schatz, and previously used to treat tuberculosis, caused by *Mycobacterium tuberculosis* [17,18]. Since then, many other antibiotic compounds have been isolated and are still now commonly used in medicine, such as tetracycline, kanamycin, and chloramphenicol, produced by *S. aureofaciens*, *S. kanamyceticus*, and *S. venezuelae*, respectively [15]. Two-thirds of commonly used antibiotics come from actinobacteria, mostly of the *Streptomyces* genus, and belong to the aminoglycosides, β-lactams, glycopeptides, lipopeptides, macrolides, and streptogramins classes [3]. Moreover, some actinobacteria not belonging to the *Streptomyces* genus also exhibit antibiotic properties, such as *Saccharothrix tamanrassetensis*, which can inhibit the growth of Gram-positive bacteria like *Staphylococcus aureus* 639c (methicillin resistant) and *Bacillus subtilis* ATCC 6633 [19]. The production capacity of actinobacteria can vary significantly, with some species producing a single antibiotic, while others generate multiple compounds and compound classes [3]. It should be noted, however, that different strains may produce the same antibiotic compound, as is the case of thiolutin, an antibiotic that inhibits *Saccharomyces cerevisiae* growth by inhibiting RNA polymerase II, produced by *S. albus* and *Sx. algeriensis* [20,21]. Although most of the chemical molecules produced by actinobacteria present antimicrobial properties, it is important to remember that they may present toxic properties. For example, chloramphenicol, produced by *S. venezuelae*, previously used to treat meningitis, plague, and typhoid fever, was prohibited for many years because of its toxicity causing fatal aplastic anemia and bone marrow suppression [22]. In the same way, valinomycin, produced by *S. fulvissimus* notably, was a powerful antimicrobial compound, but it presents an important mitochondrial toxicity [23].

##### Antifungal Compounds

Some *Streptomyces* species also produced many molecules with antifungal properties. This includes amphotericin B, a polyene macrolide produced by *S. nodosus*, used to treat candidiasis due to *Candida albicans* infections and aspergillosis caused by some *Aspergillus* species in humans and animals [24]. Amphotericin B is the antifungal substance most commonly used to treat human and animal mycoses. Another antifungal polyene, the nystatin produced by *S. noursei*, was used to treat infections due to *Geotrichum* species, notably, but also candidiasis in association with amphotericin B [25].

##### Antiparasitic Compounds

In addition to their ability to produce antibiotics and antifungal compounds, certain strains of actinobacteria are used to produce antiparasitic compounds. This is the case of *S. avermitilis*, today called *S. avermectinius*, which produces ivermectin, a drug currently used as a dewormer for farm animals and to treat certain parasitic infections in humans [26]. Additionally, tetranactin, produced by *S. aureus*, was used in Japan in the 1970s on fruit and tea crops for its miticidal properties [27]. More recently, the potential of *S. hydrogenans* strain DH16 cell-free supernatant (CFS) was described as a nematicidal agent against *Meloidogyne incognita* by inhibiting egg hatching [28].

Following these initial discoveries, research to find new molecules with medical applications became rarer due to the difficulty of discovering truly new molecules. Furthermore, although some molecules produced by actinobacteria present medical properties, these are often toxic [29]. Currently, research has shifted to identify molecules usable in the agronomic domain, mainly focusing on plant growth-promoting rhizobacteria (PGPR), natural defense stimulator (NDS), or antifungal applications.

#### 1.2.2. Agronomic Applications

Actinobacteria are well known as PGPR. There were two types of plant growth-promoting agents: microorganisms with phytostimulant properties that can promote plant growth by nitrogen fixation, phytohormone and siderophore production, and facilizing mineral intake, and those with phytoprotection activity, such as BCAs, for example [30]. The *Frankia* genus, belonging to the *Frankiaceae* family, is particularly well known and studied for its ability to live in symbiosis with actinorhizal plants, such as woody shrubs and trees, facilitating nitrogen fixation [31]. Other actinobacteria, not belonging to the *Frankia* genus, were also able to fix nitrogen. Indeed, studies have demonstrated the ability of *Corynebacterium* sp. AN1 and *Pseudonocardia dioxanivorans* to nitrogen fixation, thereby reducing the need for fertilization during cultivation [32,33]. Moreover, many actinobacteria can produce auxins, cytokinins, or gibberellins, known to promote seed germination and root growth [30]. Boukelloul et al. (2024) revealed the ability of five *Streptomyces* isolates isolated from soils of the arid Saharan region to produce siderophores, hydrocyanic acid, ammonia, and auxin indole-3-acetic acid, which promote plant growth, in addition to their activity against some phytopathogenic fungi such as *A. flavus* and *F. oxysporum* [34]. Finally, using *S. griseus* and *Streptomyces* sp. as pelleting agents on carrot and tomato seeds, respectively, resulted in increased plant growth and yield [35,36]. The ability of actinobacteria as BCAs to prevent plant diseases, caused notably by phytopathogenic and opportunist fungi, may be divided into two groups. The first group corresponds to actinobacteria that limit fungal infections by stimulating plant defenses, called NDS properties. For example, a strain of *Streptomyces* sp. called AgN23 was recently studied for its ability to reduce infection caused by *Alternaria brassicola* in *Arabidopsis thaliana* plants by increasing salicylic acid biosynthesis [37]. In the same way, a study led on *S. enissocaesilis* and *S. rochei* revealed their ability to induce plant resistance against fungal infection by *R. solani* and *F. solani* when they colonized cucumber roots [38]. The second group corresponds to actinobacteria that limit fungal infection by acting directly on the pathogen by inhibiting its growth or mycotoxin production or by degrading the mycotoxin once it has been produced. This last group will be described in another section.

## 2. Actinobacteria as Antifungal and Antimycotoxigenic BCAs

### 2.1. Various Modes of Action

**Figure 1:**
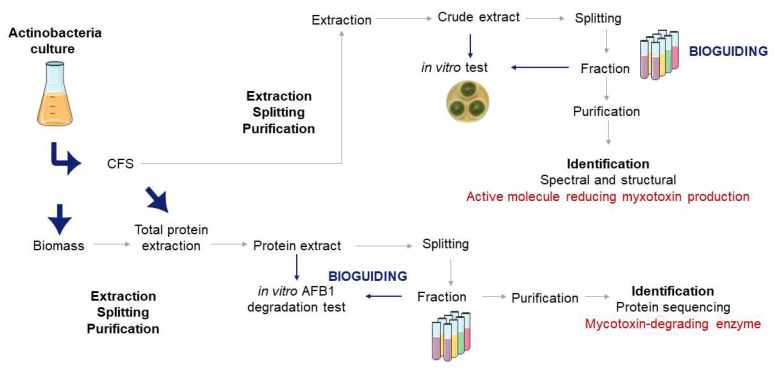
Example of flowchart to determine the mode of action of actinobacteria.

Actinobacteria have been studied for their antagonistic activities against mycotoxin-producing fungi, which can be attributed to several modes of action. Like all microorganisms used in biocontrol, actinobacteria exhibit different mechanisms to control and reduce the populations of mycotoxin-producing fungi. Confrontation is when actinobacteria commit to direct competition and confrontation with mycotoxin-producing fungi for resources and space [39]. On one hand, actinobacteria can inhibit fungal growth, preventing them from proliferating. This may involve the production of antimicrobial compounds or enzymes that degrade fungal cell walls, hindering their growth and spread [3]. They can also repress the expression of some genes involved in the secretion of toxins [40]. All these molecules may be intracellular and therefore trapped in the biomass or extracellular compounds found in the CFS. On the other hand, actinobacteria can also secrete compounds or exhibit parietal molecules on their wall that can adsorb or bind to mycotoxins. This mode of action can be defined as adsorption. In fact, by adsorbing mycotoxins, actinobacteria can reduce the bioavailability of these harmful compounds. This can be especially beneficial in agricultural and food safety applications to reduce mycotoxin concentration in crops and food products. Research was then conducted to understand the kinetics of adsorption of aflatoxin B1 (AFB1) by viable and nonviable bacteria. Although adsorption rates of AFB1 differed, both forms of bacteria successfully retained the mycotoxin [41]. Different techniques may be used to decipher the mode of action of actinobacteria. Figure 1 illustrates a possible flowchart based on a bioguiding strategy from a liquid culture of actinobacteria. In this example, active extracellular compounds are first extracted and purified from the CFS using semi-preparative high-performance liquid chromatography (HPLC) and protein precipitation. Second, active intracellular proteins are extracted, precipitated, and split. In both cases, the maintenance of activity is monitored throughout at each step using in vitro tests.

Actinobacteria are known for their enzymatic capabilities. They can produce various enzymes that can degrade or detoxify mycotoxins [42]. The detoxification mechanism corresponds to mycotoxin degradation to a less toxic compound. Some actinobacteria strains were found to degrade deoxynivalenol (DON), ochratoxin A (OTA), and zearalenone (ZEN) and reduce their concentrations to undetectable levels [43,44,45]. On a final note, they can synthesize specialized metabolites able to inhibit the growth and toxin production of mycotoxin-producing fungi. These specialized metabolites can act as antibiotics or antifungal compounds [46].

### 2.2. Actinobacteria and/or Their Specialized Metabolites Impacting Fungal Growth

Actinobacteria have yielded several novel antifungal compounds with promising properties. The first antifungal molecules produced by *Streptomyces* were discovered in the 1960s by Johnson and Dietz (1968, 1969) [47,48]. These are lomofungin [48] and kalafungin [47], respectively produced by *S. lomodensis* sp. and *S. tanashiensis* Kala, capable of reducing the growth of certain human and animal pathogenic fungi of the *Trychophyton* and *Microscporum* genera. Following these first discoveries, other antifungal molecules were identified, such as ileumycin, produced by *S. lavendulae* [49], able to reduce the growth of phytopathogenic fungi, belonging to the *Colletotrichum* genus, with a minimum inhibitory concentration (MIC) of 0.05 µg/mL. According to Barka et al. (2016), many compounds produced by *Streptomyces* species were well known for their impact on fungal growth. For example, milidiomycin produced by *Streptoverticillium rimofaciens* was very effective against *Erysiphe graminis*, also called *Blumeria graminis*, responsible for powdery mildew [3,50]. Another compound, described by Barka et al. (2016) was validamycin A, produced by *S. hygroscopicus* var. limoneus. This molecule inhibited fungal growth of *Rhizoctonia solani*, a rice pathogen, by affecting fungal metabolism after its conversion to validoxilamine A in fungal cells [3]. The growth of *R. solani* was also impacted by kasugamycin and the polyoxins B and D, produced by *S. cacaoi* var. *asoensis* and *S. kasugensis*, respectively [3]. A more recent study insight the ability of *Streptomyces* sp. 0R02 ethyl acetate extract to reduce the disease severity caused by *R. solani* of tomato plants with an MIC value of 10 mg/L [51]. Concerning another mycotoxigenic fungus, the effects of fluviricin B6, produced by *S. solisilvae*, on *Fusarium oxysporum* f. sp. *cubense* were revealed by Chen et al. (2024). Indeed, this compound was able to reduce fungal growth and spore germination with an inhibition rate of 77.52 and 80.24%, respectively [52].

Other studies have revealed the efficiency of actinobacteria co-culture in inhibiting fungal growth. For instance, studies on *S. griseoviridis* have shown its ability to limit diseases caused by *Fusarium* species on carnations and wheat when crushed into powder form and then used as a spray on the roots or as a seed coater, respectively [53]. This led to the marketing of *S. griseoviridis*, today called *Streptomyces* K61, under the name MYCOSTOP^®^ (and LALSTPOP K61 WP^®^) by Lallemand as a BCA since 2014, to combat contamination by the *Fusarium* genus and *Pythium* mainly. During the same year, Mycorrhizal Applications marketed ACTINOVATE AG^®^, composed of *S. lydicus* WYEC 108, to fight against the phytopathogens *Botrytis*, *Pythium*, *Rhizoctonia*, *Fusarium*, *Phytophthora*, and *Verticillium*. Shahid et al. (2021) revealed the ability of four actinobacteria strains (*Amycolatopsis pretoriensis* MSCA21, *Saccharopolyspora shandongensis* MSCA89, *Kribbella karoonensis* MSCA185, *S. amritsarensis* V31) to reduce the growth of *R. solani*, *Alternaria alternata*, *A. flavus*, *F. oxysporum* f. sp. lycopersici, *Sarocladium oryzae*, and *Sclerotinia sclerotiorum* (phytopathogenic fungi) with an inhibition percentage ranging from 44.8 to 90% depending on the strain [54]. Recently, a study on *S. chrestomycetius* STR-2 demonstrated its ability to reduce the growth of *Magnaporthe oryzae*, a phytopathogenic fungus of rice, with a growth inhibition of 50% compared to the control [55]. Similarly, Meliani et al. (2022) highlighted the ability of *Saccharothrix* sp. COL22 and *Actinomadura* sp. COL08 to reduce fungal growth of *A. flavus* and *F. oxysporum* f. sp. *albedinis* [56]. The co-inoculation of *F. graminearum*, one of the major DON producers, with BCAs isolated from *Streptomyces* strains on wheat grains showed promising results. Significant reductions in both fungal biomass (up to 71%) and DON levels (up to 99%) were observed. No significant reduction in DON concentration was observed when the *Streptomyces* strain was inoculated three days after the phytopathogen inoculation [57]. Another recent study revealed that the CFS of *S. exfoliates* produced after 7 days at 30 °C in starch nitrate medium allowed for the reduction in the dry weight of *A. flavus* from 70 to 94.3% when the CFS was added at concentrations from 20 to 100% (*v*/*v*) in the liquid medium. The 100% CFS concentration corresponds to *A. flavus* grown exclusively in a flask filled with CFS. *S. exfoliate* isolate was also able to reduce the sporulation of *A. flavus* on wheat seed from 92.3 to 100% [58]. More recently, biogenic silver nanoparticles produced by *Glutamicibacter nicotianae* SNPRA1 and *Leucobacter aridicollis* SNPRA2 allowed for the reduction in conidia germination of *A. flavus* and *A. ochraceus* of less than 20% compared to the control condition, at a concentration of 30 µg/mL [59]. Chimello et al. (2024) revealed the efficiency of *S. griseocarneus* in inhibiting the growth of *F. solani* in vitro by at least 20% and decreasing damage on the plant when it was inoculated on the sour passion fruit cultivar “Sol do Cerrado” [60]. Other in vivo assays led to sugar beet roots infected by *F. oxysporum*, which revealed the ability of *Streptomyces* spp. SB3-15 and SB2-23 to reduce disease severity of 80 and 93%, respectively, when seeds were treated with fermentation broth of those two strains [61].

Moreover, the effect of volatile organic compounds (VOCs) on fungal growth has recently been highlighted. For example, Wang et al. (2013) revealed that the VOCs produced by *S. alboflavus* reduce the fungal growth of *F. moniliforme*, *A. flavus*, *A. ochraceus*, *A. niger*, and *P. citrinum* with inhibition percentages ranging from approximately 60% for *F. moniliforme* to 24.8% for *P. citrinum*. Among these VOCs, the 2-methylisoborneol (2-MIB) was the most abundant, and the dimethyl disulfide completely inhibited mycelial growth and sporulation of *F. moniliforme* when it was used at a concentration of 10 µL/plate [62]. Other recent studies have highlighted the antifungal properties of VOCs, such as heptadecane, tetradecane, 3-methyl-1-butanol, acetone, and pyridine, for the most abundant, produced by *Streptomyces* spp. and *S. lavendulae* against *C. acutatum* and *Ceratocystis fimbriata*, respectively [63,64]. Finally, Boukaew and Prasertan (2020) demonstrated that L-linalool, 2-mercaptoethanol, geosmin, and heneicosane, the most abundant VOCs produced by *S. philanthi* RL-1-178 when cultivated on wheat seeds, completely inhibit the fungal growth of *A. parasiticus* TISTR 3276 and *A. flavus* PSRDC-4 on potato dextrose agar (PDA) medium. In addition, these VOCs were also effective on soybeans inoculated with the two pathogenic fungi, reducing aflatoxin concentrations to undetectable levels by HPLC (Boukaew and Prasertsan, 2020) [65]. A previous study carried out in 2018 already revealed the capacity of VOCs from *S. philanthi* RL-1-178 to reduce the incidence of anthracnose on pepper (caused by *Colletotrichum glordporioides*) to 66% compared to the control during inoculation at 5 g/L and 0% thanks to inoculation at 10 g/L. Among these VOCs, acetophenone and phenylethyl alcohol aimed to inhibit the mycelial growth and viability of five strains of chili anthracnose pathogens but remained ineffective in reducing the viability of *C. glordporioides* PSU-NY8 [66].

### 2.3. Actinobacteria and/or Their Specialized Metabolites Having an Impact on Mycotoxin Production

The *Streptomyces* bacterial genus is a prolific producer of various bio-compounds and serves as a rich reservoir for uncovering novel molecules. The activity of certain actinobacteria against the production of mycotoxins was evaluated in several studies to understand the role of the produced specialized metabolites on toxic compounds [67]. For example, a decrease in OTA concentration as a result of several strains of actinobacteria and their CFS was observed. It is thought that the examined strains can either prevent the production of OTA, degrade it, or do both [68]. Moreover, the actinobacteria strain G10, isolated from Algerian soils, was able to reduce the OTA production by *A. carbonarius* of 13.5% in PDA solid medium by reducing the expression of *acpks*, *acOTApks*, and *acOTAnrps*, genes involved in the OTA production biosynthesis pathway [69]. El Khoury et al. (2018) highlighted the ability of other actinobacteria strains (SN7, PH1, AT136, and ML5) to reduce OTA concentration from 67 to 83% without significantly impacting fungal growth by down-regulating *acOTApks* and *acOTAnrps* in *A. carbonarius* [44].

Furthermore, a notable reduction (49–71%) in deoxynivalenol (DON) levels in wheat was associated with different strains of *Streptomyces* [43,70]. It was also demonstrated that *Streptomyces* isolates were able to decrease the accumulation of a wide range of mycotoxins such as total aflatoxins, fumonisin, ZEN, T-2 toxin, alternariol (AOH), and alternariol monomethyl ether (AME) [71]. Valinomycin, cyclo(L-Pro-L-Tyr), cyclo(L-Pro-L-Val), and brevianamide F, among other metabolites produced by the *Streptomyces* strain AS1, were found to be effective in the control of mycotoxins generated by *Penicillium verrucosum* (OTA) and *F. verticillioides* (fumonisins, FUMs). Moreover, initial screening showed that the strain was successful in suppressing the mycelial growth of those species thanks to indirect interaction [72].

In the same way, the *Streptomyces* sp. AV05 strain was able to reduce both fumonisin B1 and B2 produced by *F. verticillioides* by 97.4% after 5 days in co-culture and affect the entire endo-metabolome of the fungus [73]. More recently, Strub et al. (2021) highlighted that the reduction in fumonisin B1 and B2 induced by *Streptomyces* sp. AV05—*F. verticillioides* co-culture was due to the down-regulation of genes involved in the fumonisines biosynthesis pathway [74].

Aflastatin A, produced by *Streptomyces* sp., reduced AFB1 produced by *A. parasiticus* NRRL 2999 to undetectable levels at an initial concentration of 0.5 µg/mL after 7 days at 27 °C in a liquid medium without a reduction in mycelial growth [75]. In 2015, Verheecke et al. (2015) identified three strains of *Streptomyces* (SN5, AT13, and ZL2) as negatively regulating factors of the aflatoxin cluster, notably *aflR*, *aflS*, and *aflM*, in *A. flavus*, resulting in a reduction in the AFB1 concentration to 0.2%, 2.3%, and 3.1% residual AFB1 in the medium compared to the control, respectively [40]. Furthermore, a complementary study carried out on *S. roseolus* in co-culture with *A. flavus* revealed that this strain, in addition to impacting the development of the pathogenic fungus by causing hyper sporulation, also acts on the production of AFB1 by negatively regulating all genes in the cluster (except *aflT*), leading to the reduction in AFB1 concentration to undetectable levels by HPLC [76]. The same effect was observed when *A. flavus* was grown on ISP2 medium supplemented with 1.5 g/L of *S. roseolus* CFS, allowing a reduction in AFB1 concentration under the limit of quantification after 5 and 7 days of incubation correlated with the down-regulation of the expression of the entire aflatoxin gene cluster [77]. It is important to note that among the *Streptomyces* genus, a large number of species are capable of reducing the concentration of mycotoxin and, in particular, AFB1. Indeed, according to Campos-Avelar et al. (2021), out of 59 *Streptomyces* isolates from soils and organic amendments collected in Hérault (South of France), all were capable of reducing the concentration of AFB1 during co-cultures with *A. flavus* [78]. This study is all the more interesting as it revealed that in the case of certain isolates (31 out of 59), the reduction in the AFB1 concentration was also observed when *A. flavus* was cultivated in the presence of CFS produced during 5 days at 25 °C and tested at a concentration of 10% (*v*/*v*). Likewise, a study revealed that the CFS of *S. philanthi* RL-1-178 produced for 10 days at 30 °C makes it possible to reduce the AFB1 concentration in *A. flavus* and *A. parasiticus* cultures by 96.7 and 86.7%, respectively [79].

### 2.4. Detoxification by Actinobacteria and/or Their Specialized Metabolites

In addition to their ability to act directly on the fungal development and/or its mycotoxin production, some actinobacteria can detoxify mycotoxin after its production by the fungus. For example, Teniola et al. (2005) revealed *R. erythropolis* cell’s ability to degrade AFB1 to a residual concentration of 3–6% after 72 h of incubation. Moreover, cell-free extract of this strain allowed for the reduction in AFB1 concentration of 70% after 1 h of culture at an optimal temperature of 20 °C [80]. These results were confirmed in 2006 by Alberts et al. (2006), making it possible to consider the use of these strains in biodegradation processes of AFB1 in food matrices [81]. Verheecke et al. (2015) highlighted the ability of some *Streptomyces* strains to degrade AFB1, such as ZL2, AT8, AT10, and MS1, but also AT13 and SN5, currently identified as *S. roseolus* and *S. pratensis*, respectively [40]. Other studies carried out on *R. philanthi* RL-1-178, which reduced *A. parasiticus* and *A. flavus* growth, revealed that this strain was also able to degrade 100% of AFB1 (at an initial concentration of 40 ppb) after 8 incubation days at 30 °C. Similarly, its lyophilized CFS was efficient in reducing up to 85% of AFB1 concentration after 72 h only [79]. A LC-Q-TOF MS/MS analysis of *R. philanthi* RL-1-178 highlighted two antifungal compounds: azithromycin and another unknown compound. This difference in efficiency between the cell itself and its CFS was also observed by Campos-Avelar et al. (2021). Indeed, the degradation of AFB1 in liquid medium by the 59 *Streptomyces* isolates resulted in an average AFB1 concentration corresponding to 43% of the control (control concentration = 2 µg/mL) compared to 69% in the case of the degradation assays by CFS of these isolates, revealing less effectiveness compared to the isolates themselves [78]. Due to the emergence of AFB1 contamination in Russia, Voinova et al. (2022) studied the effect of four *Rhodococcus* strains (*R. ruber* AC-180, *Rhodococcus* sp. AC-1260, *R. erythropolis* AC-1269, and AC-884) and their CFS on AFB1 accumulation in wheat. The study revealed that the four strains were able to reduce AFB1 accumulation when it was added in their culture medium (AFB1 initial concentration = 0.2 µg/mL), resulting in residual AFB1 concentrations ranging from 50 (for *R. ruber* AC-180) to 0% (for *R. erythropolis* AC-884) compared to the control condition after 24 h at 30 °C. A complementary experiment on wheat reported the ability of CFS of *R. erythropolis* AC-884 to remove 60% of AFB1 in wheat after an incubation period of 72 h [82].

Other mycotoxins, such as OTA, ZEN, and DON, can be degraded by actinobacteria. Concerning OTA, seven actinobacteria strains (AT10, AT8, SN7, MS1, ML5, G10, and PT1) were identified as being capable of degrading between 22.83 and 52.68% of OTA at an initial concentration of 0.095 µg/mL after 5 days at 28 °C [69]. A complementary study revealed that two actinobacteria strains (AT36 and SN7) were able to reduce OTA concentration in a solid ISP2 medium supplemented with 100 µg/L OTA at undetectable levels by HPLC after 5 days at 28 °C [44]. Finally, according to Campos-Avelar et al. (2020), out of fifty-nine strains of actinobacteria tested, thirty-three strains were able to completely degrade OTA in a liquid medium and only five in a solid medium. Additionally, CFSs from these strains were found to be ineffective in degrading OTA [68]. Moreover, several actinobacteria strains belonging to the *Brevibacterium* genus have been identified to reduce OTA in basal salts medium (BSM) at undetectable levels by converting it into ochratoxin α, a metabolite less toxic than OTA [83]. Another study revealed the efficiency of *Bifidobacterium longum* LA 02 and VM 14 in decreasing OTA concentration by approximately 50% in a liquid medium. The same effect was observed on patulin with *B. animalis* VM 12, which reduced patulin concentration by approximatively 80% [84].

Recently, De Troyer et al. (2024) revealed the ability of *Streptomyces rimosus* subsp. *rimosus* LMG19352 to completely degrade ZEN after 24 h at 28 °C, at an initial concentration of 5 mg/L, in vitro in LB medium and partially in MM medium after 8 days at 28 °C. Tests conducted *in planta* on wheat ears inoculated with 50 ng of ZEN per spikelet highlighted the efficiency of this strain in reducing ZEN concentration six times [85].

Finally, regarding DON, two strains of *Nocardiodes* (sp. WSN05-2 and sp. NSM2) were able to completely degrade this mycotoxin at an initial concentration of 1000 and 100 µg/mL, respectively, resulting in the formation of 3-epi-DON [86,87]. In 2002, El-Nezami et al. revealed the ability of *Propionibacterium freudenreichii* ssp. *shermanii* JS to remove some trichothecenes, such as DON, fusarenon-X, and diacetoxyscirpenol, by 18 to 93% from liquid media via an adsorption mechanism. Indeed, the absence of differences in efficiency between viable and heat-killed bacteria and the absence of degradation products observed by GC-MS support the hypothesis of mycotoxin adsorption by the actinobacteria strain [88]. More recently, the strain *Slackia* sp. D-G6, isolated from chicken intestines, was identified to be able to de-epoxidize DON into deepoxy-deoxynivalenol (DOM-1), a less toxic form of DON, at a temperature range of 37–47 °C and a pH range of 6-10 [89]. Fluviricin B6 previously cited was also able to reduce fusaric acid, beauvericin, and fusarenone by 85.28, 83.43, and 73.81%, respectively, in soil samples after 3 days of incubation at 28 °C [52].

## 3. Conclusions

In conclusion, actinobacteria, previously well known for their production of antibiotic and antiparasitic compounds, are now proving to be an infinite resource of antifungal and antimycotoxigenic compounds. All these studies highlighted the ability of many actinobacteria, and notably the *Streptomyces* genus, to prevent fungal development and mycotoxin contamination of food matrices. Furthermore, some actinobacteria were also able to detoxify foodstuffs by degrading mycotoxins. This opens the way to the use of these living microorganisms or their CFS, when effective, to ensure food safety. However, their marketing as BCAs currently remains limited due to the complexity of developing BCAs from live microbial strains and, in particular, of obtaining marketing authorization. Before considering the marketing of such products, it is essential to study their impact on the environment. However, the literature on the persistence of microbial compounds in the environment was quite poor on this subject, unlike the persistence of anthropogenic compounds, such as pesticides, and their impact on soil microbiome, which was more studied.
